# Specialized Proresolving Lipid Mediators: A Potential Therapeutic Target for Atherosclerosis

**DOI:** 10.3390/ijms23063133

**Published:** 2022-03-15

**Authors:** Juan Salazar, Daniela Pirela, Manuel Nava, Ana Castro, Lissé Angarita, Heliana Parra, Samuel Durán-Agüero, Diana Marcela Rojas-Gómez, Néstor Galbán, Roberto Añez, Maricarmen Chacín, Andrea Diaz, Nelson Villasmil, Juan Bautista De Sanctis, Valmore Bermúdez

**Affiliations:** 1 Endocrine and Metabolic Diseases Research Center “DR. Felix Gomez”, School of Medicine, University of Zulia, Maracaibo 4001, Venezuela; pirelacdaniela@gmail.com (D.P.); manuelnava_14@fmed.luz.edu.ve (M.N.); anavaleriacastro@hotmail.com (A.C.); helianapp20@hotmail.com (H.P.); nestorag17@gmail.com (N.G.); andreavalentinadiaz@hotmail.com (A.D.); nelsonvillasmilhernandez@gmail.com (N.V.); 2 Escuela de Nutrición y Dietética, Facultad de Medicina, Universidad Andres Bello, Concepción 4260000, Chile; lisse.angarita@unab.cl; 3 Facultad de Ciencias para el Cuidado de la Salud, Universidad de San Sebastián, Lota 2465, Providencia, Santiago 7500000, Chile; sduran74@gmail.com; 4 Escuela de Nutrición y Dietética, Facultad de Medicina, Universidad Andres Bello, Santiago 8370321, Chile; diana.rojas@unab.cl; 5 Departamento de Endocrinología y Nutrición, Hospital General Universitario Gregorio Marañón, 28009 Madrid, Spain; roberto_anez89@hotmail.com; 6 Facultad de Ciencias de la Salud, Universidad Simón Bolívar, Barranquilla 080002, Colombia; m.chacin@unisimonbolivar.edu.co (M.C.); v.bermudez@unisimonbolivar.edu.co (V.B.); 7 Institute of Molecular and Translational Medicine, Czech Advanced Technology and Research Institute, Palacký University, Hněvotínská 1333/5, 779 00 Olomouc, Czech Republic; juanbautista.desanctis@upol.cz

**Keywords:** specialized proresolving mediators, inflammation, resolution, atherosclerosis, intimal hyperplasia, reperfusion injury

## Abstract

Cardiovascular disease (CVD) is a global public health issue due to its high morbidity, mortality, and economic impact. The implementation of innovative therapeutic alternatives for CVD is urgently required. Specialized proresolving lipid mediators (SPMs) are bioactive compounds derived from ω-3 and ω-6 fatty acids, integrated into four families: Lipoxins, Resolvins, Protectins, and Maresins. SPMs have generated interest in recent years due to their ability to promote the resolution of inflammation associated with the pathogeneses of numerous illnesses, particularly CVD. Several preclinical studies in animal models have evidenced their ability to decrease the progression of atherosclerosis, intimal hyperplasia, and reperfusion injury via diverse mechanisms. Large-scale clinical trials are required to determine the effects of SPMs in humans. This review integrates the currently available knowledge of the therapeutic impact of SPMs in CVD from preclinical and clinical studies, along with the implicated molecular pathways. In vitro results have been promising, and as such, SPMs could soon represent a new therapeutic alternative for CVD.

## 1. Introduction

Currently, cardiovascular disease (CVD) represents the leading cause of death worldwide, with approximately 17.9 million deaths a year, translating into 44% of overall mortality rates, followed closely by cancer, respiratory diseases, and diabetes. CVD comprises heart and blood vessel disorders, and its pathophysiology is centered on either an acute or a chronic inflammatory condition [1]. Thus, CVD is marked as a serious public health concern that incurs increasingly high annual costs for the state [2,3]. 

Inflammation is a physiological immune response to pathogens and tissue damage which involves neutrophil and macrophage activation, proinflammatory cell expression, and secretions. The resolution of inflammation starts with decreasing pathogens, antigens, or any harmful agents. The decrease in trigger elements is followed by repairing damaged tissue [4]. Although simple in theory, this process involves numerous factors interacting to inhibit or dampen proinflammatory signals and trigger processes. This complexity is furthered when many proresolving mechanisms are also triggered during the inflammatory process. Some agents involved in active inflammation are functionally recalibrated to aid in the resolution process [5]. 

Unsuccessful resolution of inflammation often results in an inadequate or insufficient proresolving process. The lack of resolving factors might lead to impaired tissue repair, increased tissue damage, and a pathological chronic inflammatory state. Chronic inflammation is essential for the progression of various diseases such as atherosclerosis, obesity, cancer, chronic obstructive pulmonary disease, rheumatoid arthritis, neurodegenerative disease, among others [6]. The resolution of the inflammatory response was believed to be a passive process based on the attenuation of pro-inflammatory signaling pathways. However, the resolution of inflammation is not simply a passive restoration of homeostasis, but rather, an active process controlled by various mediators [7,8].

Among these anti-inflammatory mediators, specialized proresolving lipid mediators (SPMs) can be grouped into four families: Lipoxins (LX), Resolvins (Rv), Protectins (PD), and Maresins (MaR) [9]. Although SPMs are biosynthesized from the same precursors as proinflammatory mediators, the pathways differ markedly. These structures bind to specific receptors, restoring homeostasis by decreasing cell activation and inflammation [10]. Recently, these mediators have gained traction as a potential therapeutic target for inflammatory diseases. Studies concerning the physiology of resolution have opened new research areas from basic physiology and pharmacology to new targets for therapy [11]. 

Unresolved inflammation has been proven to play a causal role in the onset of CVD [12]. As such, a clear understanding of these endogenous inflammatory processes is critical to pinpoint the cause behind the continuous nonresolution of cardiovascular inflammation [13]. To this end, CVD research has recently focused on SPMs as bioactive compounds capable of reducing the characteristic inflammatory state that affects CVD patients [14].

SPMs have been shown to mitigate disease progression in CVD by modulating various molecular pathways which are involved in CVD pathogenesis [15,16,17,18]. This review aims to describe the role of SPMs in the inflammatory response and the molecular pathways they mediate, along with gathering and reporting evidence from cardiovascular experimental studies, specifically in the field of atherosclerosis and intimal hyperplasia. 

## 2. Specialized Proresolving Lipid Mediators and Modulation of Proinflammatory Pathways

**Figure 1:**
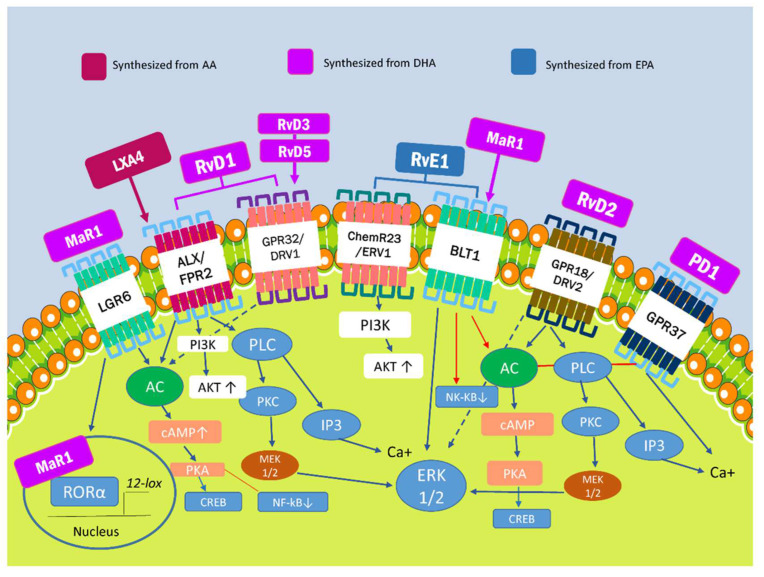
Lipoxins are derived from arachidonic acid, an ω-6 polyunsaturated fatty acid. The E-series resolvins are formed from the metabolism of the ω-3 polyunsaturated fatty eicosapentaenoic acid (EPA). Ohtter metabolites, E-series resolvins, maresins, and protectins are also formed from metabolism of the ω-3 polyunsaturated fatty acids docosahexaenoic acid (DHA). Finally, there are six known receptors belonging to the G protein-coupled receptor family for the SPMs, which are widely spread among human tissues, including leucocytes and endothelial cells. Each SPM represents a ligand for each of these receptors, frequently overlapping in matters of selectivity and biological functions. Figure 1 is presented to facilitate understanding the identified SPM receptors and the metabolic pathways involved with their proresolving biological functions, as described in murine models. AA: Arachidonic Acid. LXA4: Lipoxins A4. LXB4: Lipoxins B4. EPA: Eicosapentaenoic acid. RvE: Resolvins E series. DHA: Docosahexaenoic acid. MaR: Maresins PD1: Protectin 1. RvD: Resolvins D series. PKC: Protein kinase C. PLC: Phospholipase C. CREB: cAMP Response Element-Binding Protein. AC: adenylyl cyclase. cAMP: Cyclic adenosine monophosphate. ERK: extracellular-signal-regulated kinase. NF-κB: Nuclear factor kappa-light-chain-enhancer of activated B cells. PI3K: Phosphoinositide 3-kinase.

SPMs are the metabolites of ω-3 and ω-6 polyunsaturated fatty acids (PUFAs) generated by lipoxygenase (LOX), cyclooxygenase-2 (COX-2), and, to a lesser extent, cytochrome P450. As previously mentioned, four families of SPMs have been identified to date: lipoxins (LX), resolvins (Rv), protectins (PD), and maresins (MaR) [14]. The signaling pathways for these molecules have been described in murine models but have not yet been confirmed in human cell models (Figure 1).

It is important to highlight that the discovery of these mediators stemmed from research on animal models. In these models, injury or infection triggered inflammation along with the initmetabolic transformation of DHA and EPA into many proresolving molecules, exemplifying a novel, inexpensive approach to drug discovery compared to the traditional, costly way [19]. Most of the research was focused on animal models of myocardial injury, where a deficiency of lipoxygenase generates a shift in metabolism, cell differentiation, and leukocyte clearance. The lack of lipooxygenase favors the biosynthesis of epoxyeicosatrienoic acid and SPMs. These factors can modulate the signaling of proinflammatory pathways while favoring cardiac repair and limiting cardiac remodeling in states of acute or chronic heart failure [20,21].

The biological effects of SPMs on cells result from their binding to specific G protein-coupled transmembrane receptors (GPCRs), such as ALX/FPR2, GPR32/DRV1, ChemR23, BLT1, GPR37, and GPR18/DRV2 [22]. Overall, SPMs share some specific signaling pathways, including intracellular phosphorylation cascades and gene regulation, except for PD1, which increases intracellular calcium and triggers calcium-dependent signaling pathways instead [23]. 

The only receptor that was thought to be responsible for the biological effects of LX is formyl peptide receptor 2 (FPR2) or ALX. Besides LXA4, ALX/FPR2 is also activated by RvD1 and RvD3, whereas no receptors binding to LXB4 have been identified to date [24]. ALX/FRP2 can be found in polymorphonuclear cells (PMN), monocytes, macrophages, and T cells. Its activation by LXA4 leads to PKA activation and the phosphorylation of CREB, which stimulates the production of anti-inflammatory cytokines and macrophage polarization. RvD1 also influences the phosphorylation of AKT via the PI3K pathway, thus inhibiting NF-kB-mediated proinflammatory effects. It also enhances ERK1/2 phosphorylation via MEK1/2 to induce anti-inflammatory effects [14,25]. Recently, a new receptor called nuclear factor erythroid 2-related factor 2 (NRF2) has gained attention. It is activated by LXA4 and induces the phosphorylation of the Ser40 residue, which results in nuclear translocation. The phosphorylated NRF2 can form a heterodimer with sMAF and bind to ARE, which results in the transcription of antioxidant genes such as HO-1, NQO-1, SOD y TXN [26].

The RvD1 receptor is also a part of the GPCR family (GPR32/DRV1), which similarly binds to RvD3 and RvD5 [27]. The interaction between RvD1 and GPR32/DRV1 increases the synthesis of various miRNA in macrophages, such as miR-208a, which inhibits the transcription of the transformation suppressor programmed cell death protein 4 (PDCD4) and results in the increase of IL-10. It also increases the synthesis of miR146b, which inhibits NF-κB transcription [27]. Moreover, GPR18/DRV2 is in the same family, and is the only known RvD2 receptor. Its activation leads to the ERK1/2, PKA, or PLC pathway to promote resolution. On the other hand, GPR37 only binds to PD1, and its interaction blocks PKA and activates calcium-dependent signaling pathways which conclude in the stimulation of phagocytosis and the regulation of cytokine production [28]. 

Likewise, ChemR23 is activated by RvE1, and its downstream signaling activates AKT phosphorylation, inhibiting NF-kB inflammatory effects [29], while BLT1 binds to both RvE1 and MaR1 [30,31]. These receptors are primarily found in the bone marrow and lymphoid tissue. Also, LGR6, the human leucine-rich repeat-containing G protein-coupled receptor 6, has been reported to be a stereoselective MaR1 efferocytotic receptor [32]. Moreover, MaR1 may operate as an endogenous ligand for the nuclear receptor ROR, which regulates macrophage M2 polarization and has anti-inflammatory properties [32]. On the other hand, it was recently discovered that the GPR1 receptor, activated by RvD5n-3 DPA, upregulates cyclic adenosine monophosphate. This activation enhances phagocytosis efferocytosis [33].

ALX/FRP2, GPR32/DRV1, ChemR23, and GPR18/DRV2 expression is not limited to immune system cells; these receptors have also been identified in vascular smooth muscle cells (VSMC), in endothelial cells, and atherosclerotic lesions [34,35,36]. Furthermore, in endothelial cells incubated with DHA, immune cell adhesion and migration were reduced; this phenomenon markedly decreased when blocking RvD1 receptors (ALX/FPR2 and GPR32/DRV1). 

Altogether, these effects suggest that cell endogenous SPMs synthesis may be a paracrine pathway to decrease endothelial inflammation and maintain homeostasis in cardiovascular lesions. This effect is mediated by modulating leukocyte responses. These molecules promote migration/adhesion, reduce cell death and foam cell formation, and prolong the lifespan of both neutrophils and macrophages. All these events facilitate the clearance of infection and the resolution of chronic inflammation while reducing T cell activation and B cell antibody production, consequently decreasing the inflammatory response associated with chronic microvascular complications, cardiovascular disease, and other conditions (Table 1) [37,38]. 

Nevertheless, the role of endogenous SPMs in chronic inflammation is complex and may be influenced by several pathological and therapeutic factors that dysregulate their functions. For example, several reports from animal models have shown that obesity induced by a PUFA-rich diet, along with aging, could modify the resolving response [39,40]. This effect may be due to extended neutrophils traffic and the abundance of proinflammatory cytokine and lipid mediators in the cardioplegic and cardiorenal networks [39,40]. Molecules such as doxorubicin [41], carprofen [42], and FPR2 inhibitors [43] are capable of dysregulating immunometabolic responses by reducing SPM formation and modifying leukocyte maturation. Thus, the resolution of the chronic inflammatory process is delayed.

It is vital to highlight that most of the research on the functions of SPMs has been done on murine models, which makes it difficult to determine if one of these molecules is superior to the others in terms of modulation and biosynthesis. Moreover, these mediators are derived from different precursors and differ in their chemical structure. They also display potent receptor-mediated cell-specific actions, thus being considered a genus of endogenous molecules that act pharmacologically as immunoresolvents [10]. The characterization, purpose, interaction, and clinical utility of each family depends on the specific molecular identification of each group of receptors, their crossed reactions with other ligands such as annexin A1, and the numerous interactions within the signaling pathways [44].

MaRs appear to be the family of SPMs which is most tightly linked to macrophage-dependent cardiac tissue regeneration phenomena. They modulate the switch to a repairing phenotype which is capable of reducing the inflammatory environment by enhancing the secretion of TGF-β and decreasing the concentrations of secreted IL-6 and TNF-α [45]. On the other hand, since LXs were the first identified group from a structural and functional standpoint, it has been shown that their synthesis can be triggered by aspirin and statins. This interaction seems promising but has not been pharmacologically evaluated on a large scale [46].

Many interactions have been reported between SPMs, since they have the same cellular targets and exert functions that can coexist in terms of immunological modulation [10]. Resolvins and protectins appear early in inflammatory response. They abrogate neutrophil chemotaxis, macrophage and polymorphonuclear oxidative metabolism and stimulate the phagocytosis of damaged neutrophils [8]. Ho et al. [47] reported that both aspirin-triggered lipoxin and resolvin E1 block platelet-derived growth factor-stimulated migration of human saphenous vein smooth muscle cells and decreased phosphorylation of the platelet-derived growth factor receptor-β without altering its expression.

## 3. Role of SPMs in the Pathogenesis of Atherosclerosis

**Figure 2:**
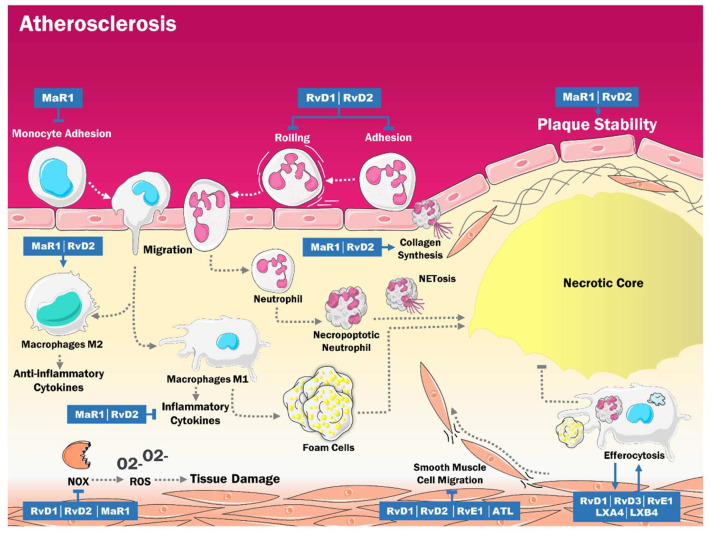
It has been shown that SPM levels are decreased in atherosclerosis, which influences the progression of the disease, given their proresolution role. These molecules decrease monocyte and neutrophil adhesion, as well as rolling. Additionally, SPM reduces proinflammatory cytokine synthesis, oxidative stress, and vascular smooth muscle cell migration while promoting plaque stability, collagen synthesis, macrophage prototype M2 activation, and efferocytosis, which reduces the size of the necrotic nucleus. ATL: Aspirin triggered lipoxins. LXA4: Lipoxin A4. LXB4: Lipoxin B4. RvD1: Resolvin D. RvD2: Resolvin D2. RvD3: Resolvin D3. RvE1: Resolvin E1. MaR1: Maresin 1. NOX: Nicotinamide adenine dinucleotide phosphate-oxidase. ROS: Reactive oxygen species. O2-: Superoxide.

Atherosclerosis is a chronic inflammatory vascular disease characterized by the thickening of the intima due to the accumulation of lipids and macrophages within a core surrounded by a layer of fibrous tissue and smooth muscle. This results in progressive vascular occlusion and the onset of thrombi at any segment of the vascular system, with clinical manifestations such as myocardial ischemia, ischemic stroke, and peripheral arterial disease [48,49]. Given its inflammatory nature, it does not come as a surprise that recent evidence associated a disbalance of lipid mediators, particularly SPMs, with the progression of the disease. When these lipids are reduced, they may favor chronic inflammation and subsequent vascular tissue damage (Figure 2) [50]. 

This phenomenon of endogenous disruption of the proresolving mechanisms is one of the singularities of the atherosclerotic process. The resolution phase involves a cessation in the recruitment of inflammatory cells, their apoptosis and clearance, and the reprogramming of macrophages to a phenotype with anti-inflammatory and tissue regenerating properties. In this regard, it has been proposed that proresolving mediators directly stimulate the regulation of PMN apoptosis, the differentiation and release of phagocytes from inflammatory sites, chemokine scavenging, and tissue repair [51,52,53]. Thus, as described in animal models of myocardial injury [40,54], aging and an obesogenic PUFA-rich diet may constitute the pathological substrate that perpetuates the chronic inflammatory process and modifies the synthesis of SPMs or other intermediate mediators that activate regulatory or resolving phenomena (Table 2).

In this sense, Viola et al. [15] performed a study with apolipoprotein E deficient mice (ApoE -/-), which have been established as the models for the study of atherosclerosis. There was a significant decrease in the levels of RvD2 and MaR1 with the progression of the disease. Furthermore, the levels of these SPMs were positively associated with the number of smooth muscle cells, collagen deposition, and the thickness of the fibrous cap. On the other hand, they were inversely related to the size of the necrotic cores. These effects can be explained by the fact that MaR1 and RvD2 increase the differentiation of M2 macrophages. They also decrease the release of proinflammatory cytokines such as TNF and IL-6 and increase the levels of TGFβ. Moreover, they improve the expression of TGFβ1 and ARG1 while reducing the expression of NOS2 in macrophages and ARG2 in endothelial cells. All these changes impact VSMC and induce collagen synthesis, contributing to atherosclerotic plaque stabilization [15,60]. 

Additionally, a study by Chatterjee et al. [55] in human tissue reported that MaR1 decreased cell–cell adhesion of monocytes and vascular cells, attenuated the activation of NF-κB in endothelial cells induced by TNF-α, and reduced the levels of cytokines and chemokines. Also, it has been proven that RvD1 and RvD2 decreased the capture, rolling, and adhesion of neutrophils. Thus, these cells have an essential role in inflammation, and among other actions, participate in the formation of the necrotic core and plaque destabilization [61,62,63]. Moreover, Cherpokova et al. studied RvD4 supplementation in mouse models with deep vein thrombosis. They reported that this supplementation could accelerate the resolution of the thrombus by stimulating the synthesis of DHA-derived SPMs within the thrombus, which may improve the phagocytosis of thrombi and apoptotic neutrophils. In addition, RvD4 reduces neutrophil infiltration and stimulates the recruitment of nonphlogistic monocytes, increasing efferocytosis [56]. 

Regarding oxidative stress (OS), another study performed in rabbits found that RvD1 decreased the levels of TNF-α-induced superoxide in vascular smooth muscle. In contrast, RvD2 reduced the reactive oxygen species (ROS) synthesis induced by the vascular lesion [57]. Furthermore, MaR1 opposed OS generated by TNF-α by decreasing the expression of NADPH-oxidases (NOX), which are the main enzymes in the synthesis of ROS in human VSMC and endothelial cells [55]. The antioxidant effects of MaR1 could be mediated by the inhibition of NF-κB signaling [64]. 

Simultaneously, there was an increase in the expression of ALX/FPR2 receptors in human atherosclerotic plaques. This receptor is considered a checkpoint in inflammation; several ligands, such as SPMs, favor the resolution of inflammation. In contrast, others, like serum amyloid A, a cardiovascular risk biomarker, are proinflammatory [65]. Hence, ALX/FPR2 signaling has an ambivalent role since it can increase the development of atherosclerosis. However, on the other hand, it can confer stability to the plaque by stimulating collagen synthesis in VSMC [60]. 

Moreover, some studies have shown that RvD1, RvD2, RvE1, and aspirin-triggered lipoxins (ATL) are all decreased in patients with atherosclerosis [66,67]. These SPMs attenuate the proliferation and migration of VSMC, probably by binding to GPR32 and ALX/FPR2, which activate cAMP/PKA signaling, a crucial pathway in cellular migration [57,68]. These findings spark some interest, given that atherosclerotic plaques are characterized by increased VSMC proliferation and migration [69]. The events could indirectly relate to the anti-inflammatory effects of disturbed flow described by Nakayama et al. [70], mediated by the adrenomedullin/G_S_-coupled receptor, a calcitonin receptor-like receptor system in endothelial cells.

All these observations relate to another study performed by Fredman et al. [71] on low-density lipoprotein receptor-deficient (LDLR -/-) mice fed with a Western diet. It was reported that advanced plaques had significantly lower levels of RvD1 and showed a slight decrease in the levels of PD1 and LXB4 while exhibiting significantly greater necrosis and higher levels of intralesional OS [72]. The same study also evidenced that in vulnerable human atherosclerotic plaques, the levels of RvD1 were significantly lower in stable plaques. This effect may be due to the increase in ROS since the calmodulin-dependent Protein Kinase I (CaMKII)-nuclear 5-LOX pathway decreases the synthesis of RvD1 [71,73]. 

Efferocytosis is a process by which cells undergoing apoptosis or necroptosis are eliminated by professional and nonprofessional phagocytic cells [74]. In atherosclerosis, there is a decrease in efferocytosis. There is a close relationship between efferocytosis and SPMs since, in macrophage cultures, the efferocytosis of apoptotic neutrophils and other microparticles increased the biosynthesis of RvD1, RvD2, and LXB4 [58]. The increase in SPMs may be mediated by the Mer tyrosine kinase (MerTK) proto-oncogene. This macrophage efferocytosis receptor increases the levels of unphosphorylated cytoplasmic 5-LOX and suppresses the activity of CaMKII [75,76]. In addition, it has been evidenced that cells undergoing necroptosis within atherosclerotic plaques may reduce the levels of SPMs, possibly leading to an impaired efferocytosis [77].

This effect can lead to positive feedback, since murine models have proven that LXA4, LXB4, RvE1, RvD1, and RvD3 regulate the functions of macrophages, stimulating efferocytosis [59,78,79]. Also, it was evidenced that RvD1 promotes efferocytosis in human macrophages. This effect is partly mediated by GPR32 and ALX/FPR2 [37]. Moreover, RvD1 decreases macrophage apoptosis after efferocytosis by reducing the ROS produced during this process via NOX inhibition by cAMP/PKA [72]. Nevertheless, within the context of atherosclerosis and particularly in unstable plaques, there is a deficiency in efferocytosis that participates in the maintenance of chronic inflammation and the growth of necrotic cores, which may be exacerbated by a decrease in SPMs levels [50].

Furthermore, even though clinical studies in this regard are scarce, it is relevant to mention the study performed by Elajami et al. [67], which had a sample of six men with coronary artery disease (CAD). Three subjects were treated with Lovaza (ω-3 fatty acids) while the other three were not. The data showed that patients with CAD had undetectable levels of LX and Rv in their serum, while healthy controls had higher and measurable levels of these markers. Also, it was reported that treatment with ω-3 fatty acids increased the levels of SPMs, being able to promote the phagocytosis of blood clots by macrophages [67]. However, this study had multiple limitations, such as the size of the sample, the absence of women in the sample, and the fact that it was not a double-blind placebo-controlled study. Hence, it is vital to carry out more clinical trials. 

On the other hand, Welty et al. [80] performed a randomized clinical trial at the Beth Israel Deaconess Medical Center in Boston, MA, with a sample of 31 subjects between the ages of 37 and 80, who had been diagnosed with stable CAD and were treated with statins. They studied the relationship between regression and progression of the atherosclerotic plaque within the coronary arteries and serum levels of ω-3 fatty acids and SPMs. It was reported that in individuals with CAD, higher serum levels of ω-3 fatty acids were associated with higher levels of RvD1 and MaR1. Furthermore, it was concluded that these SPMs might have a role in the atherosclerotic plaque regression since individuals with low levels of ω-3 fatty acids had a progression of their plaque. This phenomenon also occurred in those with high levels of ω-3 fatty acids but lo SPMs. Contrarily, those with high SPMs experienced significant regression of the plaque. 

## 4. SPMs and the Physiopathology of Intimal Hyperplasia

One of the most severe complications of atherosclerosis is vascular injury, which can be triggered by the rupture of an atherosclerotic plaque or by therapeutic interventions such as angioplasty, stent placement, or bypass surgery [81,82,83]. The body’s direct response to vascular injury consists of an inflammatory process characterized by the recruitment of leukocytes and platelets and the release of cytokines such as TNF-a, IL-1 and IL-6, ROS, and growth factors that promote the activation of VSMC [84].

**Figure 3:**
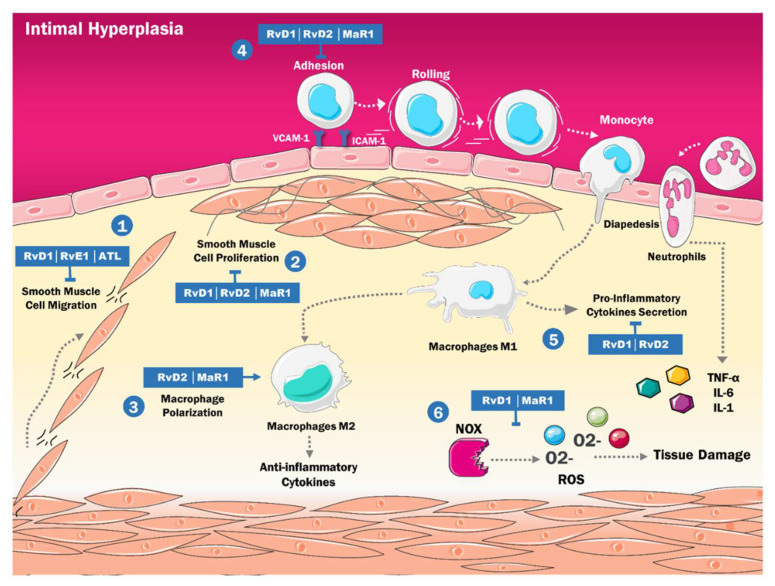
Mechanisms of actions of Specialized proresolving lipid mediators in intimal hyperplasia are involved in different processes: (1) Blocking the migration of VSMC in the intima; (2) Inhibiting the proliferation of VSMC in the intima; (3) Increasing Macrophage Polarization in the anti-inflammatory phenotype; (4) Interfering with the adhesion of neutrophils and monocytes; (5) Reducing the secretion of proinflammatory cytokines; (6) Decreasing production of ROS by NOX. IL-1: Interleukine 1. IL-6: Interleukine 6. TNF-α: Tumor Necrosis Factor Alfa. M1: Classically activated macrophages. M2: Alternatively activated macrophages. RvD: Resolvins D Serie. MaR: Maresins. RvE: Resolvins E Serie. ATL: Aspirin-triggered lipoxin. NOX: Nicotinamide adenine dinucleotide phosphate-oxidase ROS: Reactive oxygen species. O2-: Superoxide. VCAM-1: Vascular cell adhesion molecule 1 ICAM-1: Intercellular adhesion molecule 1.

The activation of these cells in the context of a proinflammatory state leads them to migrate within the blood vessel, from the media to the intima, where they proliferate and favor the synthesis of extracellular matrix. These changes lead to wall thickening and reduction in the calibre of the blood vessel in a process called intimal hyperplasia [85]. Recently, it has been reported that SPMs can reverse the inflammatory reaction triggered by vascular injury and the subsequent changes in VSMC (Figure 3) [86]. 

The main triggering factor for intimal hyperplasia is a change in the phenotype of VSMC due to the inflammatory microenvironment, which conditions their activation, migration, and proliferation. Several studies have reported that RvD1 and RvE1 can decrease VSMC migration by binding respectively to ALX/FPR2 and ChemR23, activating a cAMP-dependent signaling pathway that ultimately results in the phosphorylation of the platelet-derived growth factor (PDGF) receptor, inhibiting its action [57,68,87]. These results parallel in vitro studies in murine aortic tissue, where ATL inhibited VSMC migration via ALX/FPR2 signaling [88]. Furthermore, other murine in vitro models reported the antiproliferative effects of RvD2, RvD1 and MaR1, and how they could decrease the proliferation of VSMC to an intermediate degree [89,90].

On another note, Yang et al. [91] performed a study with murine and human aortic tissues. They evidenced a novel mechanism in promoting VSMC migration, proliferation, and inflammation in the setting of intimal hyperplasia, via neutrophil elastase (NE). This serine protease has been involved in several inflammatory diseases. This study reported that the expression of NE in VSMC increased when stimulated by atherogenic agents such as TNF-α and that NE exerted its inflammatory effects via the modulation of toll-like receptor 4 (TLR-4). At the same time, its inhibition blocked the NE-mediated dysregulation of VSMC. Moreover, studies on inflammatory diseases of the lungs [92], kidneys [93], and heart [94], have evidenced that SPMs inhibit TLR-4 signaling and reduce its inflammatory effects. This might suggest a similar protective effect in the dysregulation of VSMC in intimal hyperplasia.

Similarly, VSMC transdifferentiation has also been involved in vascular injury. Several studies have reported that VSMC can transdifferentiate into many types of cells during vascular injury via an intermediary multipotent cell that can differentiate into inflammatory cells that resemble macrophages and promote injury or fibrochondrocyte-like cells that are protective and stabilize the lesion [95,96]. SPMs might influence this process since it has been reported that PD1 [97], RvD1 [98], and LXA4 [99] promote the transdifferentiation of type I alveolar epithelial cells into type II alveolar epithelial cells, which are responsible for repairing the epithelial barrier during pulmonary vascular injury. However, the effects of these molecules on the cardiovascular sphere are still unknown, but the current results seem promising. 

Moreover, SPMs have been proven to regulate the inflammatory environment that triggers intimal hyperplasia via several mechanisms. For example, RvD1, RvD2, and MaR1 decrease the expression of adhesion molecules such as ICAM-1, VCAM-1, and E-selectin, thus regulating the interaction between VSMC and monocytes [57]. Also, in vitro models have shown decreased expression of multiple inflammatory cytokines such as IL-1a, IL-6, TNF-α, and VSMC-derived ROS in the presence of SPMs [90].

Additionally, MaR1 and RvD2 increase the polarization of macrophages into the M2 phenotype, contributing to the resolution phase of intimal hyperplasia by modifying the dominant macrophage phenotype from a proinflammatory state to a proresolving one [89]. RvE1 also mediates the same effect via its BLT1 receptor in murine models [87].

## 5. Protective Effects of SPMs during Reperfusion Injury

Reperfusion injury is a common complication in acute ischemic events such as myocardial infarction. Classically, the swift and early restoration of blood flow, known as reperfusion, has been established as the preferred treatment to prevent potential damage to myocardial tissue. However, it has been shown that, paradoxically, reperfusion may exacerbate the myocardial injury, generating necrosis and representing up to 50% of the final damage [100,101]. This results from excessive activation of neutrophils, free radicals, apoptosis and inflammatory cell infiltration, occurring once blood flow is restored. SPMs may exert a cardioprotective effect by attenuating the inflammatory process during reperfusion [102].

An in vitro study performed by Chen et al. [103] reported that LXA4 protected cardiomyocytes from reperfusion injury by promoting heme oxygenase-1 (HO-1), an enzyme known for its protective effects on hypoxic tissues. This increase in HO-1 expression seems to occur via the activation of ATP-dependent K^+^ and Ca^2+^ channels, and a decrease in TNF-a activation [104]. Moreover, LXA4 exerts an anti-inflammatory role by inhibiting NF-kB activation, thus decreasing the inflammatory cascade elicited by the release of TNF-a, IL-6, IL-8, IL-1B, and the decrease of apoptotic genes, reducing the postinfarction cardiac deterioration [103]. On the other hand, the activation of hypoxia-inducible factor-1α (HIF-1α) has also been proven to have cardioprotective effects by preventing reperfusion injury. This molecule improves mitochondrial function, decreases cardiomyocyte apoptosis, and promotes angiogenesis. Nevertheless, its mechanisms are mostly unknown [105]. 

Additionally, Zhao et al. performed a murine model and discovered that LXA4 could also inhibit the inflammatory reaction and the oxidative stress generated by reperfusion injury via other mechanisms, such as an increase in the expression of NA^+^/K^+^ ATPase. This enzyme plays a key role in regulating the membrane potential of cardiac cells. LXA4 also increases the expression of the connexin 45 protein (Cx43), which forms gap junctions between cells facilitating their electrical coupling and preventing the onset of reperfusion arrhythmias [106,107].

With regard to postinfarction cardiac repair, it is now recognized that leukocytes and macrophages initiate the acute inflammatory process and promote the resolution of inflammation. Twenty-four hours before myocardial infarction, a large number of leukocytes in the spleen increase their production of SPMs by stimulating the expression of genes that encode lipoxygenases. Then, they travel through the bloodstream towards the site of myocardial injury, where they have their resolving effects [108]. These mediators’ role in humans is still in the initial phases of discovery, with characterizations in small post-MI populations [109]. Nevertheless, the modulation of these mechanisms within the setting of heart failure, myocarditis, reperfusion injury, and even cardiac transplant appears promising. 

Moreover, the effects of ALX/FPR2 activation by RvD1 in cardiac injury have been studied. Tourki et al. [110] reported that a deficit of ALX/FPR2 in rats decreased macrophage sensitivity for inflammatory stimuli and inhibited the activation of cardiac repair markers. Furthermore, there was a decrease in the expression of lipoxygenases and SPMs, necessary for the resolution of inflammation in infarcted left ventricle tissue. At the same time, the levels of cyclooxygenases 1 and 2 were increased [110]. These results suggest that a deficit of ALX/FPR2 induces cardiometabolic disturbances and cardiac dysfunction. 

Regarding resolvins, RvD1 has been reported to have protective effects against myocardial infarction in vitro in murine models by increasing phosphorylation of the PI3K/Akt and ERK1/2 pathways. The compounds decrease the activity of caspase-3, caspase-8, and the expression of Bax, thus inactivating apoptosis pathways and increasing the levels of nitric oxide (NO) in hypoxic tissues [111]. Furthermore, there is evidence that RvD1 can reduce the size of the infarcted area and attenuate the symptoms of myocardial depression after infarction via the PI3K/Akt pathway [112,113]. 

It has been reported that RvD1, via its ALX/FPRX2 receptor, can reduce neutrophil migration and the release of proinflammatory cytokines while promoting a change from the M1 to the M2 phenotype in macrophages. This event reduces the expression of fibrosis-related genes such as COL1A1, COL2A1, and TNC, preventing postinfarction fibrosis, cardiac remodeling and structural dysfunction in the affected zone [114,115,116].

Considering this, SPMs appear to be valuable therapeutic alternatives in treating reperfusion injury by having numerous anti-inflammatory and cardio-protective effects and benefits regarding the repair and regeneration of affected tissue. Moreover, the protective effects of SPMs are not limited to the heart, having been described within the setting of ischemia in other tissues such as the brain, kidneys and liver, suggesting their potential use in situations such as myocardial infarction stroke, among others [117]. 

## 6. SPMs as Therapeutic Target in Atherosclerosis

The essential role that SPMs play in CVD inflammation resolution opens the door for new therapeutic strategies based on SPM positive regulation [118,119]. In this sense, preclinical and in vitro studies have shown how proresolution pharmacology can promote local and systemic anti-inflammation, significantly reducing the consequences of a poorly controlled inflammatory process. (Table 3).

In a rabbit model of diet-induced atherosclerosis and periodontitis, oral/local application of RvE1 significantly decreased peripheral C-reactive protein levels and inflammatory cell infiltration. This decrease parallels a reduction in vascular inflammation and atheromas [120]. Similarly, in a study with mice fed with a high-fat diet (HFD), Salic et al. [121] orally administered high and low doses of RvE1 by itself or combined with atorvastatin. The combined treatment led to a decreased transcription of pro-atherogenic genes in the aorta, a reduction of plasma levels of epoxide hydrolase 4 (an anti-inflammatory and atheroprotective mediator degrading enzyme), and the inhibition of IFN-γ and TNF-α signaling pathways. The administration of RvE1 alone or in combination with atorvastatin reduced the size of the formed atherosclerotic lesion and attenuated severe lesion development [121]. Viola et al. reported that repetitive intraperitoneal administration of RvD2 and MaR1 to HFD Apoe -/- mice prevented vascular lesions to atheroma and induced a macrophage phenotypic switch from an inflammatory to a reparative phenotype [15]. Likewise, Petri et al. conducted a study in Apoe -/- mice treated with ATL, and noted a marked inhibition in atherosclerosis progression [122].

Regarding SPMs in the resolution of the inflammatory process triggered by vascular injury, which affects vessel remodeling and permeability, some hypotheses have been proposed that pose novel therapeutic targets in CVD. Akagi et al. [89] studied the effects of intraperitoneal administration of RvD2 and MaR1 on vessel remodeling in arterial neointima formation mice models, observing a marked decrease in cell proliferation as well as in neutrophil and macrophage recruitment, with the latter favoring the M2 phenotype, eventually leading to neointimal hyperplasia attenuation. Similar results were reported by Makino et al. after administering RvD1 and PD1 isomers intravenously to rat carotid artery, balloon injury models, detecting NFkB pathway activity inhibition as a result [123]. Likewise, Miyahara et al. found a reduction in vascular cell proliferation and leukocyte recruitment, as well as neointimal hyperplasia attenuation, after local administration of RvD2 for 28 days in a rabbit model of arterial angioplasty [57]. This same group of researchers cultivated human VSMC with different doses of RvD1 and RvD2, resulting in a dose-dependent inhibition of proinflammatory gene expression, cell proliferation and migration, and monocyte adhesion [57].

Meanwhile, Liu et al. [94] studied the effects of RvE1 on vascular inflammation and neointimal hyperplasia mouse models, administered either intraperitoneally or through EPA dietary supplements and aspirin, which induce endogenous production of RvE1. Their findings suggest that combining these supplements significantly increased RvE1 endogenous biosynthesis, promoting inflammatory cell perivascular infiltration reduction and neointimal hyperplasia attenuation. RvE1 exogenous administration significantly decreased leukocyte and macrophage infiltration around the vascular lesion in a dose-dependent manner, in addition to drastically reducing neointimal formation. Conversely, Kain et al. analyzed the effect of subcutaneously administered RvD1 in its free form and RvD1 incorporated into liposomes (Lipo-RvD1) on mice subjected to coronary artery ligation. The study reported an increase in ALX/FPR2 expression, RvD1, RvD2, MaR1 and LXA4 levels, and a reduction in profibrotic gene expression and collagen deposits, leading to increased stabilization of the extracellular matrix [114].

Due to the rapid degradation of SPMs by 15-PGDH enzymes, one of the main obstacles to overcome concerning their clinical application in CVD management is to guarantee their distribution and bioavailability. This issue could be solved by increasing the availability of synthetic structures. The administration of synthetic analogues that resist rapid degradation or local compound administration through intraluminal or perivascular procedures is chemically feasible. Various researchers have developed stable synthetic analogues [99,100], nanoparticle formulations, and biodegradable RvD1-releasing devices that would allow local release of SPMs for vascular lesion modulation in animal models. This line of research has shown promising results. 

Thus, preclinical evidence positions SPMs as novel and promising mediators in the development of CVD prevention and management strategies, either by promoting SPM endogenous synthesis through PUFA supplementation as well as other molecules that also promote SPMs synthesis, or as a result of exogenous synthetic analogue administration, alone or in combination with other drugs with cardioprotective effects, such as statins. The capacity of SPMs to resolve cardiovascular, inflammatory processes marks these mediators as a possible anti-inflammatory approach to CVD management and prevention in the future.

It is essential to highlight that even though there are few studies in humans with direct quantification of these molecules, the current literature looks promising. After studying saliva samples of 254 subjects, Thul et al. [124] proposed that the RvD1:LTB4 ratio may serve as a biomarker of nonresolving inflammation and its relation to intima media thickness in cardiovascular disease. There are still no specific formulations of these molecules for diagnostic or therapeutic purposes. A study named REDUCE-IT (Reduction of Cardiovascular Events with Icosapent Ethyl–Intervention Trial) found that an EPA-only formulation lowered a composite of cardiovascular events by 25% in patients with established cardiovascular disease or diabetes mellitus and other cardiovascular risk factors [125]. Several reports have also shown that in mice fed a diet enriched in omega-3 polyunsaturated fatty acids, endogenous arachidonic acid and EPA production through desaturation of the upstream precursor omega-6 and omega-3 fatty acids, respectively, are needed to favor SPM formation [126].

On the other hand, human studies have not shown adequate levels of SPMs after dietary supplementation with fish oil in healthy volunteers [127]. Furthermore, in the study that detected aspirin-triggered SPMs in patients with diabetes and coronary artery disease, the dosage was three times higher than the conventional one. This proves that the exogenous administration of these molecules must not be dependent on their precursors. Otherwise, they would require specific metabolic and release systems [128]. 

From a molecular perspective, several reports have shown that the inhibition of leukotrienes plays a modulating role in the atherosclerotic process via the decrease of proinflammatory molecules and reactive oxygen species induced by TNF-α [129,130]. In the near future, it will be possible to consider a combined pharmacological approach of leukotriene inhibition and the induction of SPM-producing metabolic pathways as a therapeutic strategy in the management of the low-grade inflammation seen in CVD. This poses an alternative to recent trials that sought to achieve anti-inflammatory control of atherosclerotic cardiovascular disease by modulating or blocking proinflammatory signaling pathways in the final steps [131,132]. Instead, combining PUFAs (especially EPA) with SPM signaling agonists, or GLP-1 agonists with selective activators of specific SPM receptors, could constitute good pharmacological strategies destined not only to control dyslipidemia or obesity as isolated risk factors, but also with an impact on unresolved inflammation. 

To summarize, lipid mediators have been shown to be a promising alternative in the resolution of inflammation in several clinical scenarios including CVD [133,134]. However, their use in a clinical trials has been delayed by various factors, i.e., biodistribution and bioavailability, pharmokinetics and pharmcodynamics. The quick degradation of the compounds, the complex process required to significantly trigger endogenous SPM synthesis, the lack of synthetic structures that could be administered systemically, and their limited localized use pose significant problems for the use of SPMs compared to other drugs for the management of CVD. Thus, strategies that allow the systemic administration of synthetic analogues with longer half-lives are required. Further research is urgently needed in the field, given that that cardiovascular diseases are still the first cause of death worldwide.

## 7. Conclusions

Due to the increasing morbidity and mortality of CVD, describing and understanding the pathophysiology of this condition and the metabolic pathways involved in it is of the highest importance. SPMs have been identified as a potential therapeutic target in the treatment of CVD. These compounds can promote the resolution of inflammation without resulting in immunosuppression. Preclinical evidence positions SPMs as novel and promising mediators in CVD prevention and management strategies, either by promoting SPMs endogenous synthesis through PUFA supplementation and other molecules that promote its synthesis, or as a result of exogenous synthetic analogue administration, alone or in combination with other cardioprotective drugs. In addition, SPMs improve host defenses, as reported by in vitro and in vivo preclinical studies, unlike current anti-inflammatory treatments; this will likely give rise to the creation of new guidelines on the use of SPMs in CVD management. Several mechanisms are linked to SPMs, e.g., atherosclerosis in murine models and potential metabolic pathways in human diseases. However, large-scale clinical studies are required to further explore SPM analogue compounds, i.e., to test their stability and resistance to degradation, as well as their safety for human use. In addition, guaranteeing systemic bioavailability is key to any positive effects SPMs might have in vascular inflammation resolution. Regardless, the results so far have been promising, and soon SPMs could represent a novel therapeutic alternative for CVD.

## Figures and Tables

**Table 1:** Specialized proresolving lipid mediators and its actions.

SPM	Receptors	Characteristic	Actions	References
LXA4	ALX/FPR2	Synthesized from ω-6-derived from AA	-Anti-inflammatory cytokine production and macrophage polarization. -Transcription of antioxidant genes, such as HO-1, NQO-1, SOD and TXN.	[24,26]
LXB4	Not identified			
RvE1	ChemR23/ERV1, BLT1	Synthesized from EPA derived from ω-3	-Inhibition of transformation suppressor programmed cell death protein 4 (PDCD4) transcription that results in the increase of IL-10. -Inhibition of NF-kb inflammatory effects.	[25,27,28,30,31]
RvE2-6	Not identified			
RvD1	ALX/FPR2, GPR32/DRV1	Synthesized from DHA derived from ω-3		
RvD2	GPR18/DRV2			
RvD3-6	GPR32/DRV1			
PD1	GPR37	Synthesized from DHA derived from ω-3	-Stimulation of phagocytosis and regulation of cytokine production. -Mitigation of neutrophil chemotaxis and oxidative metabolism.	[23,28]
MaR1	BLT1, LGR6	Synthesized from DHA derived from ω-3	-Regulation of M2 macrophage polarization and anti-inflammatory properties. -Aid phagocytosis and efferocytosis.	[14,32]
MaR2	Not identified			

Abbreviations: AA: Arachidonic acid; EPA: eicosapentaenoic acid; DHA: Docosahexaenoic acid: LX: Lipoxins; RvE: Resolvins E serie; RvD: ResolvinsD serie; PD: Protectins; MaR: Maresins.

**Table 2:** Evidence for the action of SPMs in the pathogenesis of atherosclerosis.

SPMs	AUTHOR	MODELS	EFFECTS
RvD2 y MaR1	Viola et al. [15]	ApoE-deficient mice.	Stabilization of the atherosclerotic plaque.
MaR1	Chatterjee et al. [55]	Primary cultures of EC and VSMC from human saphen veins.	Anti-inflammatory effects in human EC and VSMC.
RvD4	Cherpokova et al. [56]	Mouse models of deep vein thrombosis.	Decrease severity of thromboinflammatory disease in vivo and improved resolution of thrombus.
RvD1 y RvD2	Miyahara et al. [57]	Rabbit models of angioplasty.	Modulation of superoxide production in VSMC.
RvD1, RvD2 y RvE2	Dalli et al. [58]	Macrophage cultures.	Efferocytosis increased SPM biosynthesis.
LXA4 y LXB4	Mitchell et al. [59]	Rodent models of peritonitits.	Significative improvement of efferocytosis.

Abbreviations: RvE2: Resolvin E2; RvD2: Resolvin D2; MaR1: Maresin 1; RvD1: Resolvin D1; EC: Endothelial cells; VSMC: Vascular smooth muscle cells; LXA4: Lipoxin A4; LXB4: Lipoxin B4.

**Table 3:** Specialized proresolving lipid mediators as a therapeutic target in atherosclerosis.

SPMs	Author	Methodology	Results
RvE1	Hasturk et al. [120]	39 rabbits with a dietary regimen for 13 weeks, were treated with topical RvE1 3 times per week.	RvE1 decreased atherogenesis and C reactive protein levels (*p* < 0.05).
	Salic et al. [121]	80 ApoE*3Leiden mice who were fed with a high-fat diet were administered different doses of RvE1.	High-dose and low-dose RvE1 reduce the size of the atherosclerotic lesion to the same degree (35%. *p* < 0.05).
	Liu et al. [94]	Male mice were fed with rodent food and treated with RvE1.	RvE1 was generated from EPA with the aid of ASA.
RvD2 and MaR1	Viola et al. [15]	ApoE -/- mice who were fed with a high-fat diet, were administered doses of RvD2 and MaR1.	RvD2 and MaR1 were signs of atheromatous plaque stability.
	Akagi et al. [89]	Smooth muscle cells from the aorta of adult male mice received an intraperitoneal injection of RvD2 and MaR1.	ASMC chemotaxis was reduced in 74% after treatment with RvD2 and MaR1.
ATL	Petri et al. [122]	Four mice who presented Fpr2 deficiency were fed with a high-fat diet for 4 weeks and later treated with ATL.	ATL blocked the progression at the root of atherosclerosis.
RvD1 and PD1	Makino et al. [123]	Male injured mice who were subjected to carotid artery angioplasty received 1 μg of RvD1 or PD1 intravenously.	RvD1 and PD1 mitigated muscle cell proliferation, as well as leukocyte infiltration.
RvD1 and RvD2	Miyahara et al. [57]	Cultures of human greater saphenous veins VSMC were isolated and treated with RvD1 and RvD2.	RvD1 and RvD2 inhibited VSMC and monocyte proliferation, migration, and adhesion.
RvD1	Kain et al. [114]	Male mice with coronary artery ligation were administered liposomes with RvD1 or Lipo-RvD1.	RvD1 decreased macrophage density.
	Orr et al. [115]	0.5 g of EOR and BDA-RvD1 were given to mice.	BDA-RvD1 was resistant to EOR and reduced neutrophil infiltration in the lungs.

Abbreviations: RvE1: Resolvin E1; RvD2: Resolvin E2; MaR1: Maresin 1; ATL: Aspirin Activated Lipoxin; Fpr2: Formyl peptide receptor 2; ASMC: Aortic smooth muscle cells; NFκB: Nuclear factor kappa B; ASA: aspirin; EPA: eicosapentaenoic acid; RvD1: Resolvin D1; RvD2: Resolvin D2; EOR: Eicosanoid oxidoreductase; BDA-RvD1: benzo-diacetylenic-17R-RvD1-methyl ester; VSMC: Vascular smooth muscle cells.

## Data Availability

Not applicable.

## Abbreviations

Cardiovascular disease (CVD); Specialized proresolving lipid mediators (SPMs); Lipoxins (LX); Resolvins (Rv); Protectins (PD); Maresins (MaR); Polyunsaturated fatty acids (PUFAs); Lipoxygenase (LOX); Cyclooxygenase-2 (COX-2); Arachidonic acid (AA); Leukotriene A4 (LTA4); 15(S)-Hydroperoxyeicosatetraenoic acid (15S-HpETE); Eicosapentaenoic acid (EPA); Docosahexaenoic acid (DHA); E-series Rv (RvE); D-series Rv (RvD); G protein-coupled transmembrane receptors (GPCRs); Formyl peptide receptor 2 (FPR2); *Programmed cell death 4* (PDCD4); Polymorphonuclear cells (PMN); Nuclear factor erythropoietin 2 related factor 2 (NRF2); Vascular smooth muscle cells (VSMC); Apolipoprotein E (ApoE); Oxidative stress (OS); NADPH-oxidasas (NOX); Reactive oxygen species (ROS); Aspirin triggered lipoxin (ATL); Low-density lipoprotein receptor (Ldlr); Calmodulin-dependent Protein Kinase I (CAMKII); Proto-oncogene tyrosine kinase Mer (MerTK); Coronary artery disease (CAD); Platelet derived growth factor (PDGF); Neutrophile elastase (NE); Toll-like receptor 4 (TLR-4); Hypoxia-inducible factor-1α (HIF-1α); Heme oxygenase 1 (HO-1); Nitric oxide (NO); High-fat diet (HFD); RvD1 incorporated into liposomes (Lipo-RvD1).
